# Conformity and Group Performance

**DOI:** 10.1007/s12110-023-09454-2

**Published:** 2023-08-05

**Authors:** Taher Abofol, Ido Erev, Raanan Sulitzeanu-Kenan

**Affiliations:** 1 Technion – Israel Institute of Technology, Haifa, Israel; 2 The Hebrew University, Jerusalem, Israel

**Keywords:** Conformity, Cultural evolution, Decisions from experience, Adaptability

## Abstract

This research provides evidence regarding the causal effect of group conformity on task performance in stable and variable environments. Drawing on studies in cultural evolution, social learning, and social psychology, we experimentally tested the hypotheses that conformity improves group performance in a stable environment (H1) and decreases performance (by hindering adaptability) in a temporally variable environment (H2). We compare the performance of individuals, low conformity groups, and high conformity groups in a four-arm randomized lab experiment (*N* = 240). High conformity was manipulated by rewarding agreement with the group’s majority and imposing a cost on disagreement. The monetary implications of conformity impaired performance in a variable environment but did not have a significant effect on performance in the stable environment. Intragroup individual-level analyses provide insights into the mechanisms that account for the group-level results by showing that lower conformity in groups facilitates efficient adaptability in the use of social information.

**Supplementary Information:**

The online version contains supplementary material available at 10.1007/s12110-023-09454-2.

What is the causal effect of conformity on the performance and adaptability of groups? Despite the number of studies devoted to conformity since Asch’s (1955) seminal work, its consequences for group performance are still contested (Kendal et al., 2018; Morgan & Laland, 2012). Theoretical models in cultural evolution suggest that in a spatially variable environment with migration between subpopulations, conformity is an effective strategy for adopting locally adaptive behavior (Boyd & Richerson, 1988; Henrich & Boyd, 1998; Nakahashi et al., 2012), whereas a temporally variant environment is predicted to select against conformity (Feldman et al., 1996; Nakahashi et al., 2012).

Although these theoretical predictions have received some support from empirical studies, they provide only tentative and partial support for their key propositions. Theoretically informed cultural-evolution studies have indeed suggested that conformity is an effective strategy for social learners in stable environments (Efferson et al., 2008; Morgan et al., 2012).^1^ Additionally, the hypothesized ill-adaptive nature of conformity in temporally variable environments is a key feature of some historical case studies, including the Pearl Harbor attack that inspired the development of Groupthink theory (Janis, 1972). However, these studies cannot directly assess causal relationships between conformity and group performance and adaptability since they provide only correlational and qualitative historical evidence. Importantly, to the best of our knowledge, no previous studies in this field have experimentally treated group conformity.

Drawing on this literature, we hypothesize that conformity improves group performance in a stable environment (H1) and decreases performance in a temporally variant environment (H2). Specifically, our second hypothesis states that conformity reduces group adaptability. We utilize an experience-based decision-making task (Erev & Roth, 2014; Hertwig et al., 2004) that includes both stable and variable environments (Rakow & Miler, 2009) and accommodates group decision-making. Importantly, our design includes a novel treatment of group conformity, allowing us to directly estimate the effect of conformity on group performance in temporally stable and variable environments. In addition to treating group conformity, the experiment also includes two conditions in which the same task is performed by individuals and by memory-assisted individuals to identify the unique effect of a group (versus an individual) on performance and to assess a competing theoretical explanation. The result is a four-arm randomized lab experiment (*N* = 240) with *Low Conformity (LoConf) Group*, *High Conformity (HiConf) Group*, *Individual*, and *Memory-Assisted Individual* conditions. All participants play a computer game consisting of 100 sequential choices between two alternatives, presented as two unlabeled buttons on a computer screen. Unknown to the participants, the game had two stages: in stage one (first 60 rounds) one option dominated the other, and in the subsequent stage (the last 40 rounds), the other option becomes dominant. Following the advice of Morgan and Laland (2012), the experimental design and analysis control for subjects’ asocial information in conjunction with social information when forming their decisions, allowing us to verify that the treatment indeed enhanced the impact of social information independently from asocial information.

Our findings provide support for the hypothesis that conformity decreases group performance in a temporally variable environment (H2). Although high-conformity groups performed better than low-conformity groups in the stable stage of the game, this difference was statistically insignificant; thus our findings do not support the hypothesis that conformity increases group performance in stable conditions (H1). Intragroup individual-level analyses provide further insight into the mechanisms that account for the group-level results. In the altered environment, social information becomes less influential within low-conformity group members, whereas it retains a strong impact on high-conformity group members. These results imply that low conformity within groups facilitates greater adaptability in the use of social information. When social information is useful (in a stable environment), low-conformity groups allot only slightly less weight to social information than high-conformity groups. However, faced with indications of a change in the environment, low-conformity group members tend to reduce the weight they assign to social information, whereas high-conformity groups maintain (and even slightly increase) the level of decision weight for this information, despite its poor informative quality. In the following sections, we report the experimental study conducted to test these hypotheses.

## Experimental Design

We utilize an experience-based decision-making task (Erev & Roth, 2014; Hertwig et al., 2004) that includes both stable and variable environments (Rakow & Miler, 2009) and accommodates group decision-making. A similar experimental design was employed by Lejarraga et al. (2014) to study the performance of groups and individuals in decisions from experience under stable and changing environments. Each participant faced six 100-round games, in one of two conditions: Individual and Group. In the Group condition, the participants sat in a triad in front of a single computer screen and had to reach a decision. Groups performed better than the average individual under stable conditions, yet group performance was slower to recover from a change in the decision environment. Lejarraga et al. (2014) explained these findings by alluding to the superior memory of groups compared with individuals, a quality which rendered groups less adaptive. However, group dynamics and the processes that led to the decisions within each group were not gauged nor recorded. We therefore posit that this experimental design does not exclude the possibility that the results were caused by conformity, a potential quality of groups but not of individuals. Specifically, conformity (rather than memory) provided an advantage to groups over individuals in the stable stage of the game and undermined groups’ adaptability after the change in the game.

To test our hypotheses, we employ one of the games used by Lejarraga et al. (2014) and included a novel treatment of group conformity, allowing us to directly estimate the effect of conformity on group performance in temporally stable and variable environments. In addition to these two main conditions, the experiment also includes two conditions in which the same task was performed by individuals and by memory-assisted individuals to identify the unique effect of a group (versus an individual) on performance, and to assess a competing explanation of enhanced memory.

Two hundred and forty university students participated in the study. Participants were randomly assigned to the *Individual* condition (*n* = 30), *Low-conformity* (LoConf) group condition (*n* = 90, *n*_groups_ = 30), *High-conformity* (HiConf) group condition (*n* = 90, *n*_groups_ = 30), or the *Memory-assisted individual* condition (*n* = 30). All the participants took part in a clicking paradigm experience-based decision task (Erev & Haruvy, 2016; Erev & Roth, 2014) with 100 rounds. The specific task was based on game 5 in Lejarraga et al. (2014). In each round, participants clicked on one of two buttons and received feedback consisting of their obtained payoff (from the selected button) and the forgone payoff (the payoff that they could have received if they had selected the other button). In all rounds, one button had a higher expected value than the other, and the aim was to maximize the number of points obtained over the 100 rounds of the game.

**Fig. 1:**
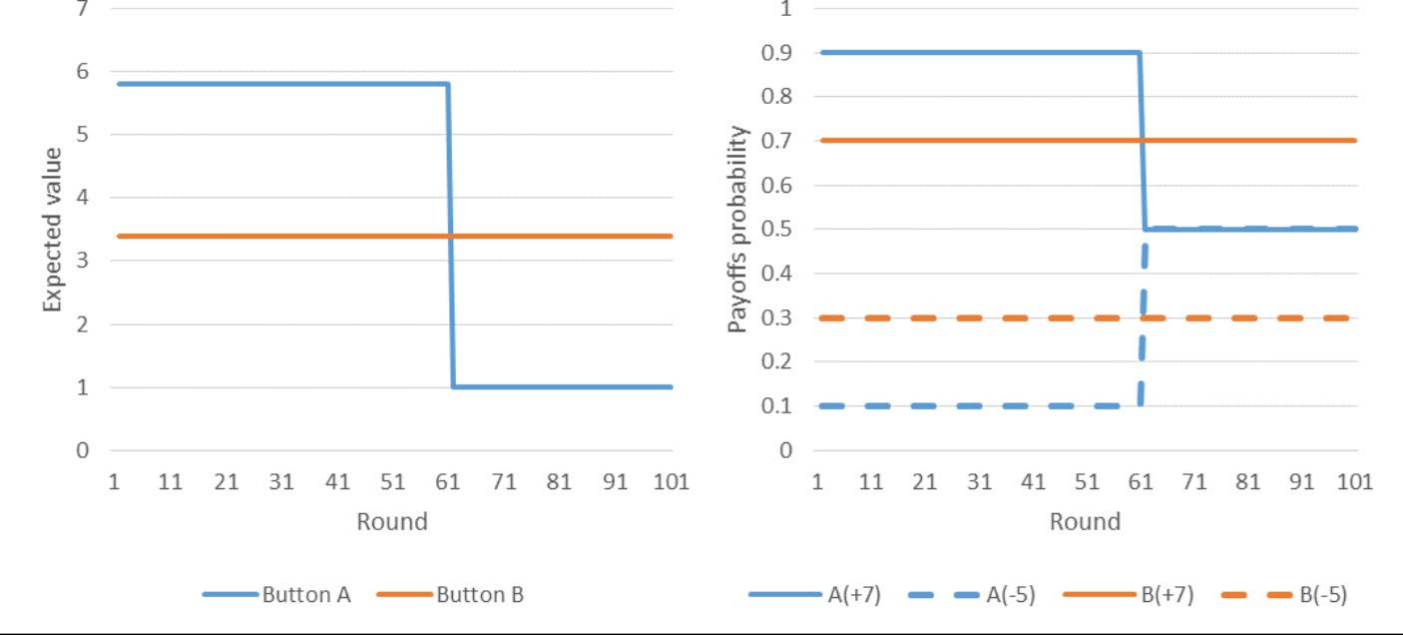
The experience-based task. The left panel presents the expected value of the two buttons throughout the game. Up to round 60 the higher paying button is A (blue), whereas from round 61 onward, the dominant button is B (orange). The right panel presents the probability of obtaining a positive (+ 7; solid lines) and a negative (− 5; dashed lines) payoff, in each of the two buttons across the 100 rounds

The temporal change in the environment was simulated by implementing two stages in the clicking task, as is described in Fig. 1: In the first 60 rounds (“stable environment”) of the game, one button dominated the other, and in rounds 61–100 (“altered environment”) the relationship between the two options was reversed. Specifically, in the stable environment the two keys were randomly assigned to two prospects: 7 with *p* = 0.9 and − 5 otherwise (EV = 5.8), and 7 with *p* = 0.7 and − 5 otherwise (EV = 3.4). After 60 rounds, the probability of gaining 7 by choosing the dominant key dropped to 0.5 (EV = 1), thus its expected value in rounds 61–100 was lower than that of the alternative key, which became dominant in this stage. In the stable environment, participants were able to learn which of the two keys obtained a higher payoff on average (expected value); however, the reduction in the expected utility of the dominant option key from round 61 onward rendered this learned information obsolete, thus requiring participants to identify the change, relearn, and adapt in order to maximize their payoff.

### Procedure

Participants assigned to the individual condition were provided with asocial information only in the form of their payoff for each decision round. In the two group conditions, participants were provided with both asocial information (decision payoff) and implicit social information. They inferred the latter from whether their individual choice was a minority opinion or aligned with the majority in each round, based on whether it mismatched or matched the group’s choice, respectively.

All participants performed the experiment individually on their own computer terminal. In the individual condition, participants read the following instructions:

You will play a game of 100 rounds. In each round, you will be asked to choose one of two money machines. When you click on the machine, you will win or lose points. Your payoff at each round will be determined based on your choice and the probability of winning, which may change during the game. At the end of each round, you will see your payoff and the forgone payoff had you chosen the other machine. If you have any questions, please ask the experimenter. Please press start when you are ready.

In the two group conditions, we deviate from the design of Lejarraga et al. (2014), in which group participants sat together by a single computer terminal and only their collective choice in each round was recorded. To gain better control over intragroup mechanisms, specifically the social information obtained by each group member in each round, each group member sat individually by her/his computer terminal and interacted with the two other group members only via the game interface. Specifically, in each round every participant was asked to make a choice and to wait for the other players’ choices. After all group members had completed their choices, each participant was informed of the group decision (based on majority rule) and his/her payoff given this choice. The software recorded these interactions between group members—both individual (group member) and group decisions. Participants were also informed of the forgone payoff each player would have received had the group chosen the other key.

The instructions of the low conformity (LoConf) group condition were as follows:

You are part of a group of three players. You will play a game of 100 rounds. In each round, you will be asked to choose one of two money machines. When you click on the machine, you will win or lose points. Your payoff at each round will be determined according to your choice and the other players’ choices and to the probability of winning, which may be changed during the game. At the end of each round, you will see your payoff and the forgone payoff had the group chosen the other machine. If you have any questions, please ask the experimenter. Please press start when you are ready.

The LoConf game has multiple Nash equilibria.^2^ Under the simplifying assumption that the players know which button provides a higher expected value (EV), the game has five pure strategy equilibria. In the first equilibrium, all three players select the high EV button. Three additional equilibria are asymmetric: in these equilibria, exactly two players select the high EV button, and the third player selects the low EV button. The fifth equilibrium is weak and inefficient: all three players select the low EV button. This inefficient outcome is an equilibrium because no player can benefit from deviating unilaterally, and it is weak because deviating is not costly.

In the high-conformity (HiConf) group condition, the procedure and instructions are the same as for the low-conformity (LoConf) group condition, with one difference: the payoff for each player is also affected by whether their individual choice aligns with the majority choice. In the case of a minority opinion, two points are deducted from the dissenting participant’s payoff, whereas each majority participant receives an additional point.^3^ This alteration has two implications: it eliminates the three asymmetric equilibria and enhances the stability of the two consensual equilibria. In the HiConf game, both the efficient and inefficient consensual equilibria (wherein all players select either the high or low EV button) are strong as unilateral deviation is costly. In summary, these multiple equilibria entail that any behavior can be considered rational under certain beliefs about the behavior of the other players, under both levels of conformity.

When players cannot know with certainty which button maximizes their payoff, behavior in the present game is likely to depend on the way the players have adapted to past experiences. As noted above, the common assumptions in cultural evolution predict that conformity facilitates efficiency in a stable environment but may impair maximization in a dynamic environment. The following experiment is designed to evaluate the robustness of these predictions.

Beyond its direct effect on players’ potential payoff, this novel treatment of conformity is intended to draw their attention to social information—specifically, whether the player’s opinion aligns with or differs from the majority. We therefore test the efficacy of this treatment at both the group and individual levels. At the group level, we estimate the prevalence of minority opinions within group decisions across the two group conditions. At the individual level, we assess whether the treatment increases players’ propensity to change their choices after finding themselves in a minority opinion, controlling for the monetary payoff. These analyses are reported in the “Results.”

Cultural-evolutionary theory identifies several important considerations in designing experimental studies of conformity. First, not all forms of social learning constitute conformity. Social learning is defined as the acquisition of behavior through observation or interaction with other individuals (Aplin et al., 2017; Rendell et al., 2011), in contrast to asocial learning, which is based on personal experience. Conformity occurs when social learning leads to the homogenization of group behavior through the disproportionate adoption of popular traits (Efferson et al., 2008). Second, since the use of social information increases as asocial information becomes costlier and the task more difficult (Morgan et al., 2012), conformity is only expected in cases where a group member is naive regarding how to cope with the task. Third, in order to distinguish between the effects of asocial and social information, the former must be controlled for (Morgan & Laland, 2012).

The design of this study addresses these considerations. First, given that conformity leads to the homogenization of group behavior, we evaluate the efficacy of our conformity treatment by comparing the proportion of minority decisions [i.e., 1 – *P*(consensus)]. Second, participants are naive regarding the task and receive both social (majority/minority opinion) and asocial (noisy payoff) information, which are recorded for each group decision (participants in the individual condition received only asocial information). Third, our design creates an equally difficult task in the three conditions, thus leading to the same propensity to rely on social information in the two group conditions (see Kendal et al., 2018:652–53). Lastly, following the advice of Morgan and Laland (2012), the experimental design and data analysis control for asocial information, which subjects process in conjunction with social information when forming their decisions. This allows us to verify that the treatment indeed enhanced the impact of social information independently from asocial information.

The two group conditions provide the comparisons required to test our main hypotheses in both the stable and variable stages of the game. In order to address the alternative claim made by Lejarraga et al. (2014), according to which the differences found between individuals and groups resulted from the enhanced memory of groups, we included a fourth condition: “memory-assisted individual.” In this condition participants played the same game as in the individual condition with one difference: the results of all the previous trials were shown in two lists on the screen. Each list included the payoffs received when choosing each button, providing participants with a “perfect memory” of the payoff history.

The instructions in all conditions informed the participants that a change in the probability of gaining the positive payoff is possible, without indicating how prevalent the change would be, nor when in the sequence of trials it would occur. Our goal was that participants would not assume a static environment. In all three conditions, payoffs in points were converted to monetary sums. In the group conditions, all three members received full compensation; thus group members had the same economic incentives as individual participants. The mean total individual compensation was equivalent to US $9.70.

### Statistical Analysis

To identify the effect of conformity on performance in a temporally varying environment, we estimate the interaction effect of change and conformity on performance. Equation 1 presents this relationship:

1 $$\begin{array}{l}{\rm{Logit}}\left[ {{\rm{E}}\left( {{Y_{ir}}{\rm{ = 1}}} \right){\rm{|}}{{\left( {{\rm{Round}},{\rm{Change}},{\rm{Condition}}} \right)}_{ir}}} \right]\\{\rm{ = }}\,{\upbeta _{\rm{0}}}\,{\rm{ + }}\,{\upbeta _{\rm{1}}}\,{\rm{roun}}{{\rm{d}}_{\rm{i}}}{\rm{ + }}\,{\upbeta _{\rm{2}}}\,{\rm{chang}}{{\rm{e}}_{ir}}{\rm{ + }}\,{\upbeta _{\rm{3}}}\,{{\rm{C}}_{{\rm{indi}}{{\rm{v}}_{\rm{i}}}}}{\rm{ + }}\,{\upbeta _{\rm{4}}}\,{{\rm{C}}_{{\rm{H}}{{\rm{C}}_{\rm{i}}}}}{\rm{ + }}\,{\upbeta _{\rm{5}}}\,{\rm{chang}}{{\rm{e}}_{ir}} \times {{\rm{C}}_{{\rm{H}}{{\rm{C}}_{\rm{i}}}}}{\rm{ + }}\,{\upbeta _{\rm{6}}}\,{\rm{chang}}{{\rm{e}}_{ir}} \times {{\rm{C}}_{{\rm{indi}}{{\rm{v}}_{\rm{i}}}}}{\rm{ + }}\,{\varepsilon _{ir}}\end{array}$$

where $Y$ is a binomial variable that represents a choice of the higher expected-value option (Maximization) [*Y* = 1] or not [*Y* = 0], by group or individual $i$ in round $r$, conditional on game round (1–100), the stage of the game [stable (1–60) or altered (61–100)], and experimental condition (individual, LoConf group, or HiConf group). The two coefficients of interest are β_4_ and β_5_, which represent the difference in performance between HiConf and LoConf groups in the stable stage (rounds 1–60), and the difference in the effect of change on performance between HiConf groups and LoConf groups (the latter being the reference condition), respectively. Given that choices are clustered within groups/individuals, Eq. 1 was estimated using generalized estimating equations (GEE) (Liang & Zeger, 1986) with a logit-link function, and standard errors are clustered within groups. Statistical analyses were performed using the Stata 17 software.^4^ The data and code for this study are available at 10.7910/DVN/ANGHVJ.

## Results

### Assessing the Validity and Efficacy of the Conformity Treatment

We begin by assessing the validity and efficacy of our conformity treatment. To assess the efficacy of the conformity treatment at the group level, we compare the propensity for minority opinions within group decisions in the LoConf group and HiConf group conditions. The proportion of group decisions involving the minority opinion was overall higher among the LoConf group condition (61.5%) than the HiConf group condition (37.72%; *N* = 5980; χ^2^ = 338.23; *p* < 0.001). Moreover, in line with the definition of conformity, in the HiConf condition group decisions grew increasingly homogeneous, as reflected by the decreasing proportion of minority opinions (see Figure A1 in the ESM).

To assess whether the conformity treatment serves as a priming mechanism that draws attention to social information, we estimate its effect on the individual = level propensity to change a player’s choices after finding themselves in a minority opinion, controlling for the monetary payoff. Table A1 presents this analysis in the ESM. The results show that the two sources of information influence choices in the expected way since payoff negatively affects the propensity to change one’s choice, and holding a minority opinion positively affects this choice. Importantly, these findings show that the effect of social information (being in the minority) has a stronger effect on the propensity to change the subsequent choice among group members in the HiConf condition, controlling for asocial information. These results attest to the validity of the conformity treatment since it increases the effect of social information on participants’ choices beyond the effect of payoff.

### Descriptive Results

**Fig. 2:**
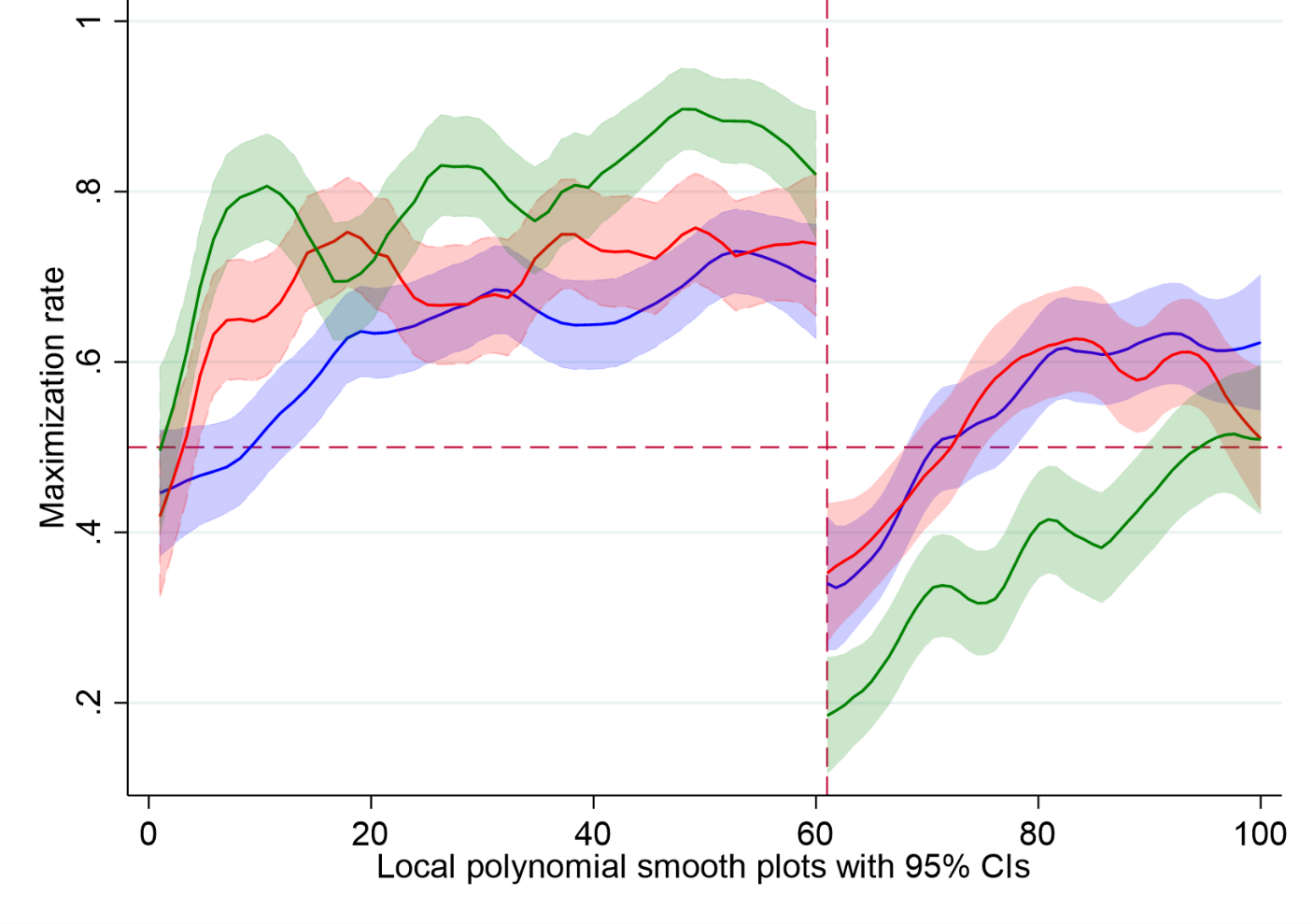
The probability of choosing the maximizing choice before and after the change (round = 61) and across conditions (blue: individual; red: LoConf group; green: HiConf group). The vertical dashed red line indicates the point of change in the game

Figure 2 presents the probability of choosing the maximizing option across individuals (blue), LoConf groups (red), and HiConf groups (green) over the course of 100 rounds. It is apparent that, in the initial part of the game, both group conditions were quicker to learn which is the maximizing choice, and HiConf groups continued to improve in making the correct choices, beyond the level of individuals and LoConf groups. However, after round 60 this pattern reverses as HiConf groups appear to be slowest in adapting to the change in the game. In fact, even at the 100th round (40 rounds after the change) they only reach about 50% probability of correct choices.

Table 1 reflects these results by presenting the average probabilities of a maximizing choice in the stable (rounds 1–60) and altered (61–100) stages of the game. Although HiConf groups obtain the highest average probability of correct choices in the stable stage (0.79), their performance was the worst among all four conditions in the altered stage (0.37). Notably, the change in the parameters of the game resulted in a decline in the performance of both individuals and groups across conditions, yet the biggest decline is evident in the performance of HiConf groups.

**Table 1:** Average probabilities of correct choice

Condition	Average maximizing choice$| \text{r}\le 60$	Average maximizing choice$| \text{r}>60$	Difference
Individual	0.627	0.543	−0.09
LoConf group	0.694	0.535	−0.16
HiConf group	0.792	0.372	−0.42
Memory-assisted Indiv.	0.701	0.598	−0.10
^χ2^	118.83	134.89	
*p*-value	< 0.001	< 0.001	

### Generalized Estimating Equation Results

To formally estimate the varying effect of environmental change on performance across group conformity levels, we conducted a set of generalized estimating equation (GEE) analyses, reported in Table 2. The dependent variable in all models is a binomial variable that takes the value 1 if the dominant option was chosen (maximizing choice), and zero otherwise. Model 1 is a simple preliminary model that estimates the overall effects of the change in the game, and the number of rounds played. The effect of the number of rounds played is positive and statistically significant, providing support for a learning process. The effect of the change in the game is negative and statistically significant, reflecting a sharp decrease in performance after the change.

**Table 2:** Generalized estimation equation (GEE) analyses of group performance

	Model 1	Model 2	Model 3	Model 4
Variables	Learning & change	Conformity & change (1–80)	Conformity & change (1–90)	Conformity & change (1–100)
**Change × HiConf group**		−1.344*	−1.321*	−1.199*
		** (0.628)**	** (0.603)**	** (0.599)**
Change **×** Individual		0.234	0.287	0.343
		(0.579)	(0.556)	(0.547)
Change	−1.984***	−1.697***	−1.699***	−1.752***
	(0.324)	(0.506)	(0.495)	(0.489)
Round	0.021***	0.020***	0.021***	0.021***
	(0.003)	(0.003)	(0.003)	(0.003)
Individual		−0.308	−0.309	−0.309
		(0.349)	(0.350)	(0.350)
HiConf group		0.528	0.529	0.529
		(0.363)	(0.364)	(0.364)
Constant	0.265*	0.243	0.206	0.203
	(0.123)	(0.253)	(0.249)	(0.250)
Observations	8,980	7,200	8,091	8,980
Number of groups	90	90	90	90

Coefficients represent logit estimates. Group clustered standard errors in parentheses; *** *p* < 0.001, ** *p* < 0.01, * *p* < 0.05

Models 2–4 fit Eq. 1 to the data, including the stable stage and the 20, 30, and full 40 rounds after the change, respectively. These separate analyses allow us to address the fact that as time elapses since the change, the game reverts to a new stable state. The coefficients of the interaction between change and conformity level are presented first (in bold). All three estimates are negative and statistically significant, suggesting that the negative effect of change on performance is greater for HiConf groups than for LoConf groups (the reference category). In contrast, the coefficients of the interaction between individuals and LoConf groups (Change × Individual) are small and statistically insignificant, suggesting that the effect of change on the performance of individuals and LoConf groups is not significantly different.

Note that the GEE results indicate no significant differences between the performance of LoConf and HiConf groups in the stable stage of the game (rounds 1–61), given the insignificant coefficients of *Individual* and *HiConf group*. These results do not provide support for H1. Replacing the reference group with “individual” permits a comparison of HiConf groups and individuals in the stable and altered stages, as shown in Table A2 in the ESM. This analysis shows that HiConf groups performed better than individuals in the stable stage (*p* = 0.013) and worse in the altered stage (*p* = 0.005). These results seem to replicate those of Lejarraga et al. (2014); however, our findings suggest that the different performance levels of individuals and groups in stable and variable environments should be attributed to group conformity since such differences were not found when comparing individuals with low conformity groups. Moreover, Table A2 shows no significant differences between individuals and memory-assisted individuals, providing no support for the proposition that enhanced memory accounts for the different performance levels of individuals and groups in stable and variable environments.^5^

**Fig. 3:**
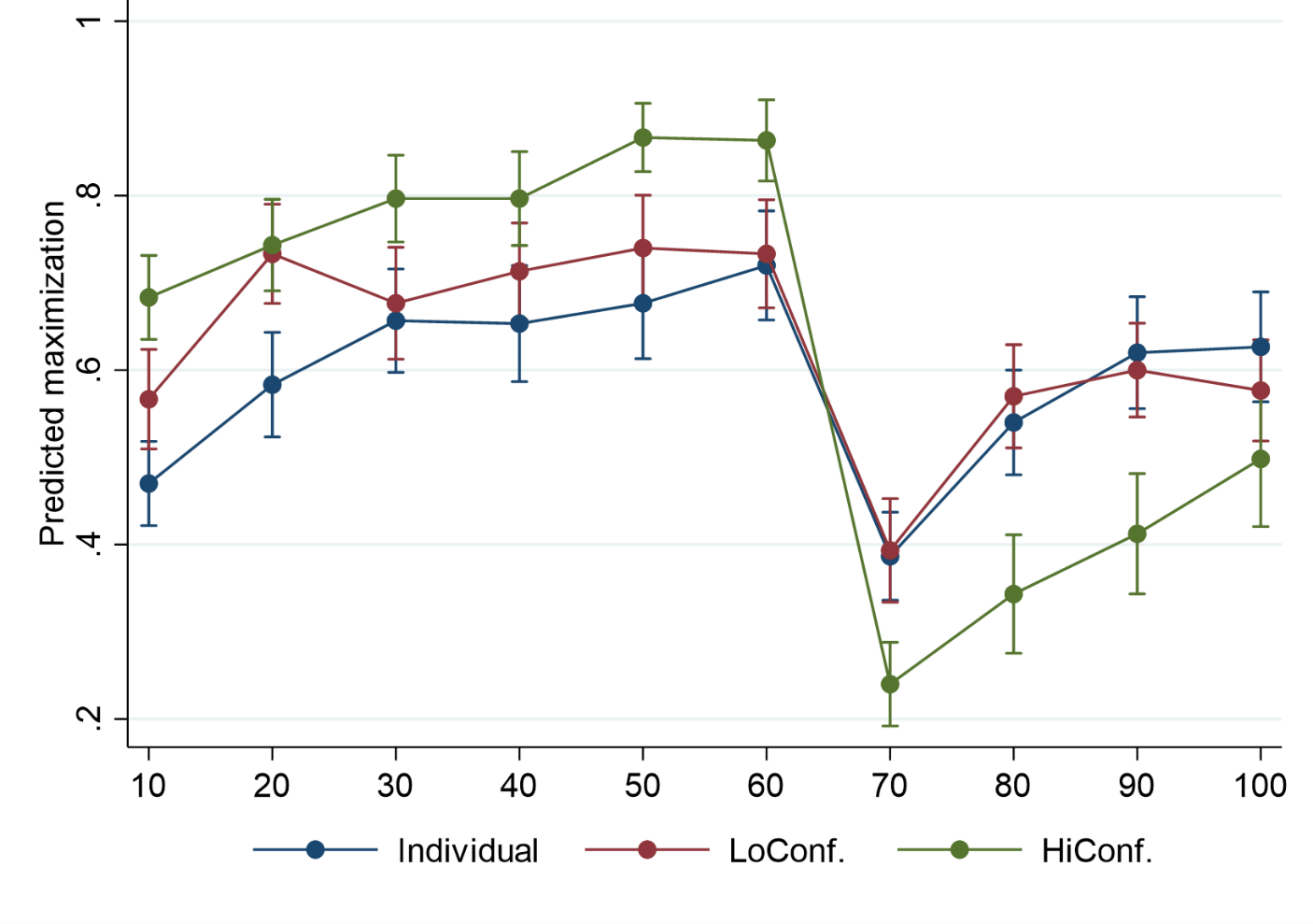
Mean performance across conditions throughout the game. GEE estimates for 10 sets of 10 rounds (CI = 1SE)

Figure 3 graphically presents the GEE estimates of performance over each set of 10 rounds and across conditions. Point estimates are accompanied by 1 SE confidence intervals. The performance of HiConf groups is higher than that of LoConf groups and individuals, but only the latter differences are statistically insignificant. However, the decline in performance due to the change in the game is more pronounced in the case of HiConf groups for each of the three sets of 10 rounds after the change. In the final 10 rounds of the game, this difference diminishes to a statistically insignificant level. Note that individuals and LoConf groups similarly adapt to the altered environment. A similar graph, including the memory-assisted individual condition, is shown in Figure A2 in the ESM.

### Individual Level Within-Group Mechanism

In order to obtain a better understanding of the group-level results, we conducted a set of (within-group) individual-level analyses, to estimate the role of social information—holding a minority opinion—in determining the decisions of group members: namely, whether they change their choice in the subsequent round. Following the advice of Morgan and Laland (2012), the analysis also includes asocial information—the payoff received—in the respective round. Note that the payoff in the game is determined by the group decision; thus, at the individual level its informative content is conditional on whether a player’s recent choice is in line with the group or is a minority opinion. These analyses therefore estimate the effect of both social and asocial information on the propensity that a group member would change her/his choice with respect to the choice in the previous round.

**Fig. 4:**
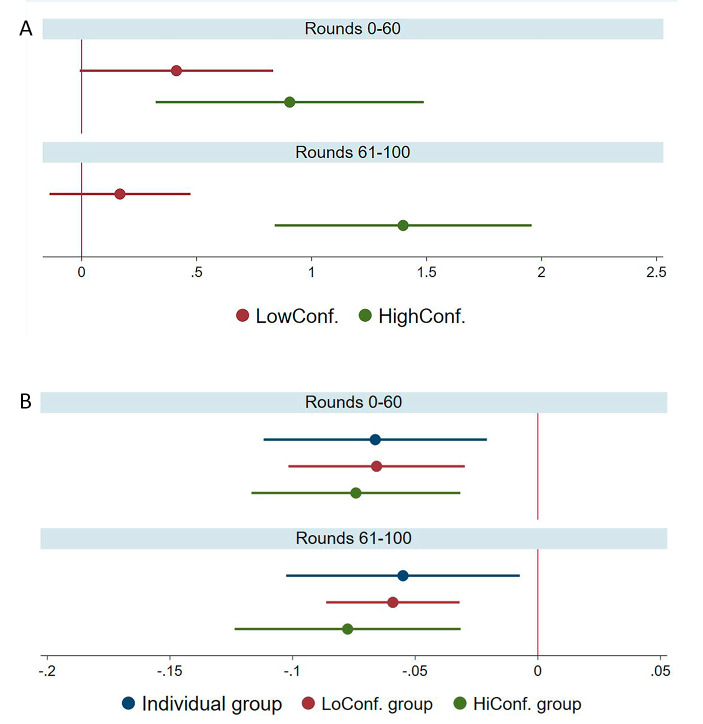
(**A**) Estimated effects of social information (being in the minority) on a player’s propensity of to change her/his choice in the subsequent round, across group conditions in the stable stage of the game (1–60), and after the change (61–100). (**B**) Estimated effect of asocial information (payoff, when being in the majority) on a player’s propensity to change her/his choice in the subsequent round, across the three experimental conditions in both stables of the game. Estimates represent logit coefficients with 95% CIs

Table 3 presents GEE estimations of the propensity to change one’s subsequent choice. The main findings are depicted in Fig. 4. Models 5 and 6 include an interaction term between payoffs and minority opinions to allow the effect of the payoffs to vary across situations where a player’s choice aligns or does not align with the group choice. The models also include interactions between social information and group condition, between the payoff and group condition, and a three-way interaction between payoff, minority opinion, and group condition.

**Table 3:** GEE estimation of group members’ choice change

	Model 5	Model 6
Variables	Stable stage	After change
Social info. (minority opinion)	0.409	0.167
	(0.212)	(0.155)
Asocial info. (lagged Payoff)	−0.0643***	−0.0591***
	(0.0183)	(0.0138)
Payoff × minority opinion	0.0599*	0.0854***
	(0.0240)	(0.0241)
HiConf condition	−0.114	−0.471
	(0.238)	(0.253)
Social info. × HiConf condition	0.516	1.232***
	(0.369)	(0.323)
Lagged payoff × HiConf condition	−0.0111	−0.0184
	(0.0285)	(0.0271)
Lagged payoff × Minority opinion × HiConf condition	0.0263	−0.0164
	(0.0397)	(0.0353)
Round	−0.0056*	−0.0003
	(0.0025)	(0.0033)
Constant	−0.848***	−0.964**
	(0.137)	(0.314)
calculated estimates:		
Social info. in HiConf	0.925**	1.399***
	(0.302)	(0.283)
Asocial info. in HiConf	−0.075***	−0.078***
	(0.022)	(0.023)
Asocial info. when in minority (LoConf)	−0.004	0.026
	(0.018)	(0.018)
Asocial info. when in minority (HiConf)	0.022	0.010
	(0.037)	(0.029)
Observations	10,502	7,097
Number of groups	60	60

Logit estimates with group clustered standard errors in parentheses; *** *p* < 0.001, ** *p* < 0.01, * *p* < 0.05

Both social and asocial information were found to predict an individual’s likelihood of changing their subsequent choice, but these effects vary across group conditions and stages of the game. In the stable stage (rounds 1–60: Model 5), social information (holding a minority opinion) increases the likelihood of changing one’s subsequent choice. This effect is smaller and marginally significant in the LoConf group condition (*b* = 0.409, *p* = 0.054), compared with the effect in the HiConf group condition (*b* = 0.925, *p* = 0.002), controlling for asocial information (payoff interacted with minority opinion). However, this difference in the effect of social information during the stable stage is not statistically significant (*p* = 0.163). The change in the game is followed by contrasting changes in the role of social information in the two group conditions. Although the effect of social information diminished in the LoConf group condition (*b* = 0.167, *p* = 0.282), it increased in the HiConf condition (*b* = 1.399, *p* < 0.001), and the difference in its effect between the two group conditions is statistically significant in this phase of the game (*p* < 0.001), as shown in Fig. 4B.

In both stages of the game, asocial information (receiving a higher payoff) decreased the propensity of players to change their subsequent choice, as expected. This effect was identified only when players’ choices aligned with the group. Instead of a reversed effect in the event of being in the minority, the effect became small and statistically insignificant. This pattern was similar for both group conditions and did not significantly alter across the two stages of the game. Furthermore, the effect of asocial information on majority group members was not significantly different from its effect on players in the individual condition. These results are depicted in Fig. 4B.

These findings provide an individual-level account for the varying group-level adaptability under high and low conformity. Whereas asocial information in our setting is a noisy yet unbiased signal that facilitates learning and adaptation, social information is based on accumulated learning and therefore reflects *collective*, *lagged, asocial-based knowledge*. Once players have the opportunity to experience the game and learn to evaluate the options, social information becomes beneficial because the majority is less likely to err. However, this advantage of social information becomes a drawback in an altered environment where lagged information is rendered obsolete. This can be demonstrated by the estimated likelihood that a minority opinion would be correct (a maximizing choice) over the course of the two stages of the game, as shown in Figure A2 in the ESM. In the stable stage, as players gain experience, the probability that a minority opinion is correct becomes significantly lower than 0.5, indicating that the majority is more likely to be correct. However, right after the change in the game, a minority opinion is more than 60% likely to be correct because the majority opinion reflects outdated knowledge of the environment. At this stage, social information is nonadaptive.

Given this varying utility of social information in stable and altered environments, the finding that social information becomes less influential among members of LoConf groups when the environment changes, whereas it increases its influence among members of HiConf groups (Fig. 4A), accounts for the reduced adaptability of HiConf groups.

## Discussion

This research provides evidence regarding the causal effect of conformity on group performance in stable and variable environments. Drawing on studies in the evolution of culture and the social transmission of information (Boyd & Richerson, 1988; Henrich & Boyd, 1998; Kendal et al., 2018; Nakahashi et al., 2012), we experimentally test the hypotheses that conformity improves group performance in a stable environment and decreases performance (adaptability) in a temporally variant environment.

Our experimental design builds on the works of Rakow and Miler (2009) and Lejarraga et al. (2014) and extends them by introducing a conformity treatment, randomly assigned to half of the groups (HiConf) and not to the rest (LoConf), and by fully controlling and recording intragroup choices and interactions. In line with Morgan and Laland (2012), we assess the efficacy of the conformity treatment by comparing the proportion of minority decisions; assign participants to an experience-based decision-making task, in which they are naive; and expose them to both social and asocial information, which are recorded for each group and individual decision. Lastly, the equal difficulty of the task across experimental conditions creates an equal baseline propensity to rely on social information in the two group conditions.

The results do not provide support for the hypothesis that conformity increases group performance in stable conditions (H1). High conformity groups did perform better in this stage, but the current analysis does not permit rejecting the null hypothesis (*p* = 0.146). Our findings provide support for the hypothesis that high conformity negatively impacts group performance in a temporally variable environment (H2). This statistically significant result was retained for ~ 30 rounds following the change in the environment, before diminishing in the final 10 rounds as the game effectively reverted to a new stable environment.

Individual-level analyses within groups provide further insights into the mechanisms that account for the group-level results. In a stable environment both asocial (payoff) and social (minority/majority opinion) information appear to influence behavior. Notably, the two appear to exert a similar influence on the choices of individuals in the two group conditions (social influence is more influential on members of high conformity groups, but this difference is statistically insignificant at this stage of the game: *p* = 0.197). However, in the altered environment, social information became less influential within low-conformity group members, while it retained a strong impact on high-conformity group members. This difference likely accounts for the reduced adaptability of high-conformity groups. Asocial information in our setting is a noisy yet unbiased signal that equally facilitates learning in both stable and temporally variable environments. Social information integrates noisy asocial information and therefore reflects collective, lagged, asocial-based knowledge. Given the opportunity to experience a stable environment over time, social information thus becomes increasingly beneficial since the majority is less likely to err than individuals. Yet, as our empirical results show, this particular cumulative and lagged quality of social information becomes a drawback in an altered environment, and a minority opinion enjoys a greater likelihood of being correct than the majority, limiting the adaptiveness of social information.

Specifically, the individual-level analyses suggest that low conformity within groups facilitates greater adaptability in the use of social information. When social information is useful (stable environment), low conformity groups allot similar (though somewhat lower) weight to social information as high-conformity groups. However, faced with indications of a change in the environment, low-conformity group members tend to allocate less weight to social information, whereas high-conformity groups maintain the same level of decision weight for this information.

To the best of our knowledge, these findings are the first to provide human behavioral evidence for the causal effect of conformity on the performance and adaptability of groups. These findings support evolutionary models of social transmission of information (Boyd & Richerson, 1988; Henrich & Boyd, 1998; Nakahashi et al., 2012), particularly the claim regarding the limited adaptability of conformity in a temporally variable environment, thus contributing to the debate over the adaptability of conformity (Kendal et al., 2018; Morgan & Laland, 2012).

The results of this research correspond to the findings of Lejarraga et al. (2014) but demonstrate that the different patterns of performance of individuals and groups in stable and temporally variable environments are more likely due to group conformity than to group memory. High-conformity groups performed better than individuals in stable environments and relatively worse than individuals after the change in the game. However, these differences were not found when comparing individuals with low-conformity groups, which is arguably the missing condition in the Lejarraga et al. (2014) study. We suggest that the high-conformity condition in this study is a better replication of the group condition in Lejarraga et al. (2014) than our low-conformity condition because there is more pressure for conformity when one is sitting with a group face-to-face than when interacting over the computer (the LoConf condition). We also did not find differences in performance when comparing individuals with those equipped with a memory-assisted feature. As far as individuals are concerned, however, our findings should be considered in conjunction with those of Rakow and Miler (2009), who found that memory-aided individuals were less adaptive to change in five out of ten decision problems (in two experiments), and had a nonsignificant effect in the other games.

### Electronic Supplementary Material

Below is the link to the electronic supplementary material.

Supplementary Material 1

## Footnotes

1 Experimental studies with human subjects that relied on these cultural-evolution models have concentrated on identifying conditions that facilitate conformist behavior (Efferson et al., 2008; McElreath et al., 2005; Morgan et al., 2012; Toelch et al., 2010), which are less relevant to the current research.

2 Nash equilibria constitute a profile of choices from which no agent is motivated to deviate unilaterally.

3 The feedback informed the players of the majority choice, the payoff from the majority choice, the conformity payoff, and the payoff from the forgone majority choice. For example: “The group majority chose ‘left.’ You were in the majority (chose ‘left’). Your payoff is − 4: −5 (for group choice) + 1 (for the majority opinion). Had the group chosen ‘right,’ the payoff for the group choice would be 7.”

4 The main question addressed in this study pertains to the role of conformity in the performance and adaptability of groups; thus most analyses focus on this, sometimes excluding the memory-assisted individual condition, for clarity purposes. This latter condition was included to address an alternative explanation for the different performance of individuals and groups and thus is of secondary importance. This condition is fully included in the analysis in the “Results” and in the ESM.

5 Memory-assisted individuals, however, generally performed better than individuals throughout the game, in both the stable and variable stages, but this difference is not statistically significant (*p* = 0.114).

## References

[CR1] Aplin, L. M., Sheldon, B. C., & McElreath, R. (2017). Conformity does not perpetuate suboptimal traditions in a wild population of songbirds. *Proceedings of the National Academy of Sciences*, *114*(30), 7830–7837. 10.1073/pnas.1621067114 PMC5544276 28739943

[CR2] Asch SE (1955). Opinions and social pressure. Scientific American.

[CR3] Boyd, R., & Richerson, P. J. (1988). *Culture and the evolutionary process*. University of Chicago Press.

[CR4] Efferson C, Lalive R, Richerson PJ, McElreath R, Lubell M (2008). Conformists and mavericks: The empirics of frequency-dependent cultural transmission. Evolution and Human Behavior.

[CR5] Erev, I., & Haruvy, E. (2016). Learning and the economics of small decisions. *The handbook of experimental economics* (Vol. 2, pp. 638–716). Princeton University Press.

[CR6] Erev I, Roth AE (2014). Maximization, learning, and economic behavior. Proceedings of the National Academy of Sciences.

[CR7] Feldman MW, Aoki K, Kumm J (1996). Individual versus social learning: Evolutionary analysis in a fluctuating environment. Anthropological Science.

[CR8] Henrich J, Boyd R (1998). The evolution of conformist transmission and the emergence of between-group differences. Evolution and Human Behavior.

[CR9] Hertwig R, Barron G, Weber EU, Erev I (2004). Decisions from experience and the effect of rare events in risky choice. Psychological Science.

[CR10] Janis IL (1972). Victims of groupthink: A psychological study of foreign policy decisions and fiascoes.

[CR11] Kendal RL, Boogert NJ, Rendell L, Laland KN, Webster M, Jones PL (2018). Social learning strategies: Bridge-Building between fields. Trends in Cognitive Sciences.

[CR12] Lejarraga T, Lejarraga J, Gonzalez C (2014). Decisions from experience: How groups and individuals adapt to change. Memory & Cognition.

[CR13] Liang KY, Zeger SL (1986). Longitudinal data analysis using generalized linear models. Biometrika.

[CR14] McElreath R, Lubell M, Richerson PJ, Waring TM, Baum W, Edsten E, Efferson C, Paciotti B (2005). Applying evolutionary models to the laboratory study of social learning. Evolution and Human Behavior.

[CR15] Morgan T, Laland K (2012). The biological bases of conformity. Frontiers in Neuroscience.

[CR16] Morgan, T., Rendell, L. E., Ehn, M., Hoppitt, W., & Laland, K. N. (2012). The evolutionary basis of human social learning. *Proceedings of the Royal Society B: Biological Sciences*, *279*(1729), 653–662. 10.1098/rspb.2011.1172 PMC3248730 21795267

[CR17] Nakahashi W, Wakano JY, Henrich J (2012). Adaptive social learning strategies in temporally and spatially varying environments. Human Nature.

[CR18] Rakow T, Miler (2009). Doomed to repeat the successes of the past: History is best forgotten for repeated choices with nonstationary payoffs. Memory & Cognition.

[CR19] Rendell L, Fogarty L, Hoppitt WJE, Morgan TJ, Webster MM, Laland KN (2011). Cognitive culture: Theoretical and empirical insights into social learning strategies. Trends in Cognitive Sciences.

[CR20] Toelch U, Bruce MJ, Meeus MTH, Reader SM (2010). Humans copy rapidly increasing choices in a multiarmed bandit problem. Evolution and Human Behavior.

