# Supplementary material for: Conformity and Group Performance
Source: Hum Nat. 2023 Aug 5;34(3):381–99. doi: 10.1007/s12110-023-09454-2 (PMC10543786; doi:10.1007/s12110-023-09454-2)
Supplement: Supplementary file 1 — Supplementary Material 1 [file 12110_2023_9454_MOESM1_ESM.pdf]

Electronic Supplementary Material

Conformity and Group Performance

Taher Abofol
Technion – Israel Institute of Technology
taheraf@gmail.com

Ido Erev
Technion – Israel Institute of Technology
erev@tx.technion.ac.il

Raanan Sulitzeanu-Kenan*
The Hebrew University
raanan.s-k@mail.huji.ac.il

*Corresponding author

Human Nature 34(3), 2023, https://doi.org/10.1007/s12110-023-09454-2

Figures and Tables

Figure A1. GEE estimate of group decisions with minority opinion throughout the game and across the two group conditions 3
Figure A2. Predicted performance across conditions throughout the game 4
Figure A3. The probability that a minority opinion is correct (a maximizing choice) throughout the game, across the two group conditions 4

Table A1: Generalized estimation equation (GEE) of individual choice-change. 2
Table A2: GEE analyses of performance across all four conditions 3
Table A3: Comparing the experimental effects under a nuanced analysis of the HiConf condition. 5

To assess the validity of the conformity treatment, the following analysis estimates the propensity of group members to change their choice, given their asocial (payoff) and social (minority opinion) information in the previous round. Importantly, this analysis is intended to assess whether social information is more influential in the high-conformity condition, controlling for asocial information. Models 1 and 2 present the effects of the two sources of information on group members in the low-conformity and high-conformity conditions, respectively. It is evident that the two sources of information influence choices in the expected way, as payoff negatively affects the propensity to change one's choice, and holding a minority opinion positively affects this choice. However, while the effects of asocial information in the two conditions are similar in size, the effects of social information are stronger among group members in the high-conformity condition.

Model 3 provides formal comparisons of the effects of asocial and social information in the two conditions, by including observations from the two conditions, and estimating the interactions between each of the information sources and experimental conditions (HiConf). These results show that the difference in the effects of asocial information in the two conditions is not statistically significant (p=.558), while the difference in the effects of social information is (p=.029).

Table A1: Generalized estimation equation (GEE) of individual choice-change.

	Model A1
Lagged minority opinion	.274 (.153)
Lagged payoff	-.066 (.015)***
HiConf	-.287 (.238)
Lagged minority opinion × lagged payoff	.077 (.018)***
**Lagged minority opinion × HiConf**	**.898 (.317)****
Lagged payoff × HiConf	-.004 (.024)
Minority opinion × payoff × HiConf	-.011 (.030)
Round	-.0026 (.0012)*
Constant	-.860 (.147)***
Observations	17,599
Number of groups	60

Coefficients represent logit estimates. Group clustered standard errors in parentheses; *** p<0.001, ** p<0.01, * p<0.05.

Figure A1 presents the proportion of group decisions which included minority opinions throughout the game and across the two group conditions. This probability is expected to be 0.75 under random individual choices; however, given asocial learning, this probability is likely to decline. While the mean proportion of minority opinions in the LoConf condition remains around 0.6 throughout the game, this proportion in the HiConf condition steadily decreases along the stable stage of the game until it reaches a nadir of 0.25, just before the change in the game. This proportion rises to around 0.4 after the game change, but remains lower compared to the LoConf condition.

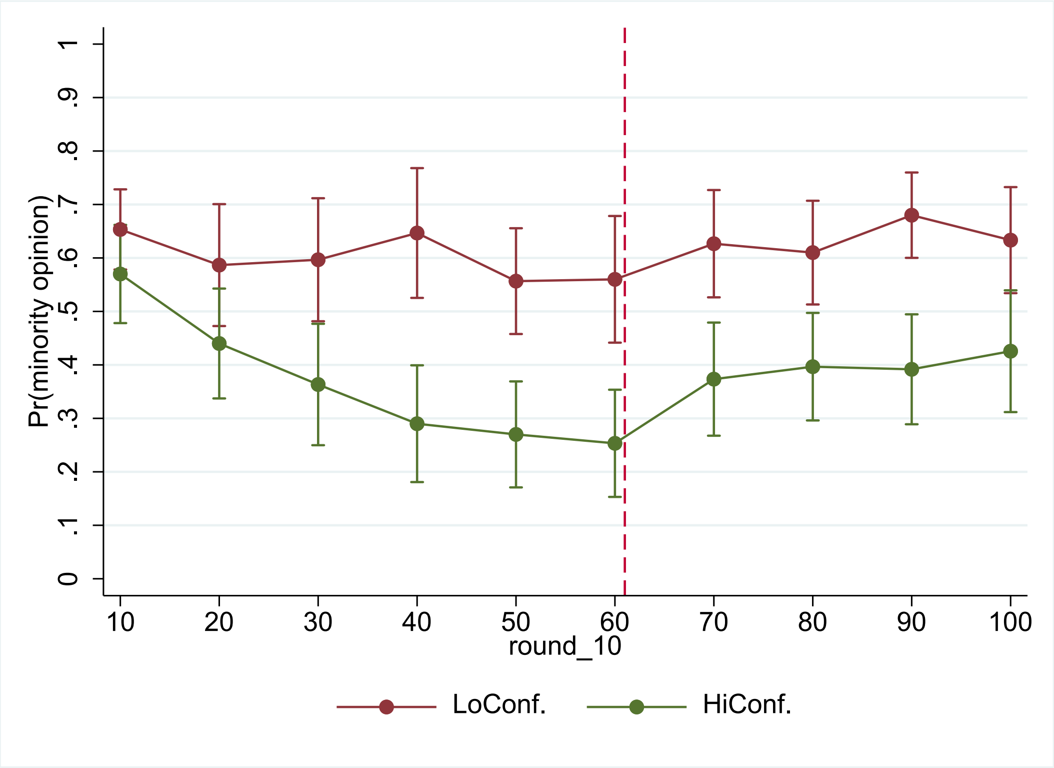

Figure A1. GEE estimate of group decisions with minority opinion throughout the game and across the two group conditions (CIs=95%). The vertical dashed red line indicates the point of change in the game.

Table A2: Generalized estimation equation (GEE) analyses of performance across all four conditions, with 'individual' as reference category.

VARIABLES	Model 1 Learning & change	Model 2 Conformity & change (1-80)	Model 3 Conformity & change (1-90)	Model 4 Conformity & change (1-100)
Change × HiConf group		-1.580**	-1.609**	-1.545**
		(0.562)	(0.546)	(0.545)
Change × LoConf group		-0.235	-0.287	-0.344
		(0.579)	(0.555)	(0.547)
Change × (Individual +memory)		-0.143	-0.205	-0.121
		(0.464)	(0.448)	(0.435)
LoConf group		0.309	0.310	0.310
		(0.349)	(0.350)	(0.351)
HiConf group		0.837*	0.839*	0.841*
		(0.338)	(0.339)	(0.339)
Individual + memory		0.344	0.345	0.346
		(0.302)	(0.303)	(0.303)
Change	-1.930***	-1.490***	-1.436***	-1.470***
	(0.266)	(0.404)	(0.399)	(0.396)
Round	0.0218***	0.0202***	0.0214***	0.0222***
	(0.00261)	(0.00269)	(0.00267)	(0.00262)
Constant	0.227*	-0.0836	-0.118	-0.141
	(0.101)	(0.213)	(0.213)	(0.214)
Observations	11,980	9,600	10,791	11,980
Number of groups	120	120	120	120

Coefficients represent logit estimates. Group clustered standard errors in parentheses; *** p<0.001, ** p<0.01, * p<0.05.

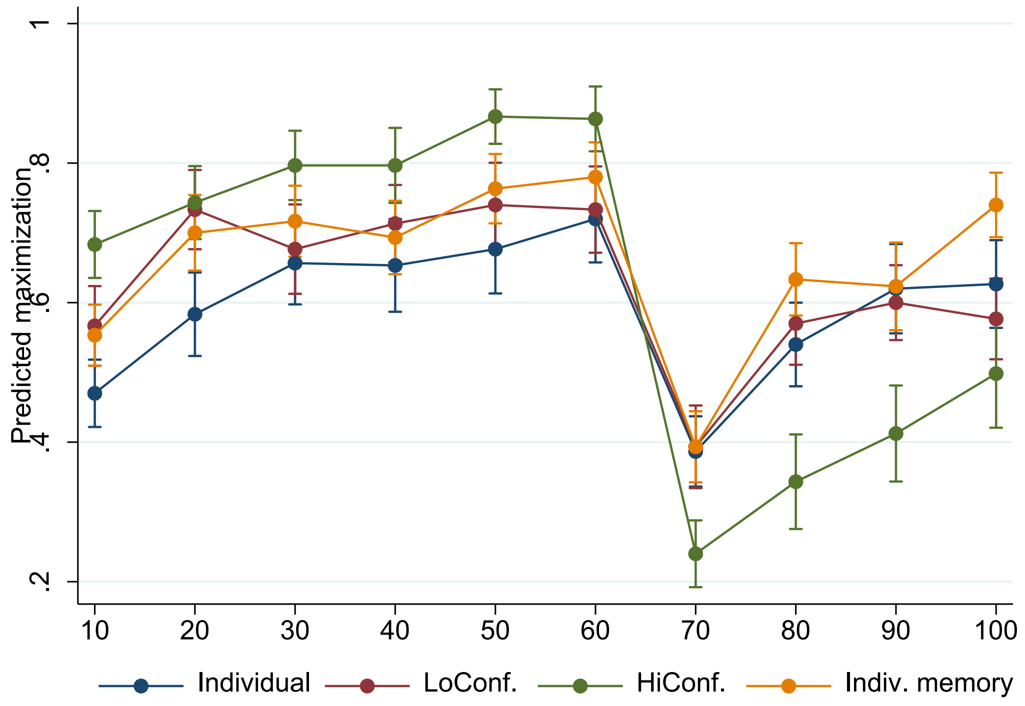

Figure A2. Predicted performance across conditions throughout the game. GEE estimates for 10 sets of 10 rounds (CI = 1SE).

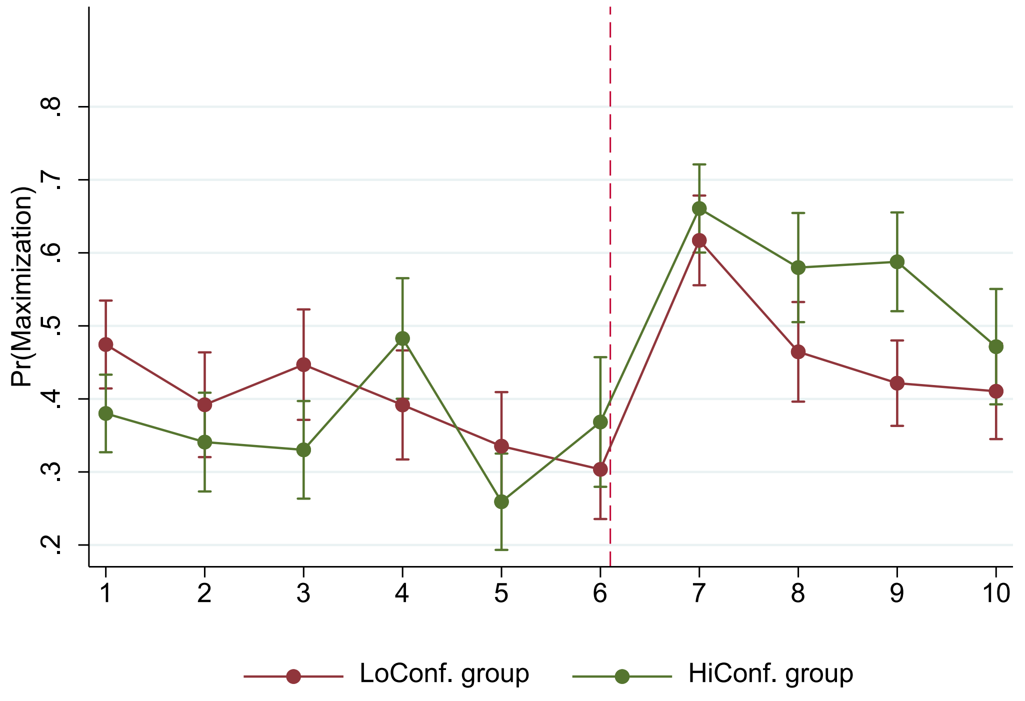

Figure A3. The probability that a minority opinion is correct (a maximizing choice) throughout the game, across the two group conditions (CI = 1SE). Estimates are based on a logit regression with group clustered SEs. The vertical dashed red line indicates the point of change in the game.

Table A3: Comparing the experimental effects under a nuanced analysis of the HiConf condition.

VARIABLES	(1)<br>LoConf	(2)<br>HiConf	(3)<br>HiConf (Nuanced social info.)
Minority	0.412	0.914**	1.270***
	(0.215)	(0.300)	(0.307)
Majority			1.048***
			(0.197)
Lagged payoff	-0.0658***	-0.0748***	-0.0877***
	(0.0185)	(0.0218)	(0.0200)
Lagged payoff × Minority	0.0595*	0.0861**	0.0983***
	(0.0241)	(0.0320)	(0.0295)
Lagged payoff × Majority			0.0157
			(0.0207)
Change	0.0717	0.0861	-0.186
	(0.124)	(0.174)	(0.223)
Change × Lagged payoff	0.00666	-0.00230	-0.0119
	(0.0137)	(0.0207)	(0.0252)
Change × Minority	-0.245	0.496*	0.558*
	(0.209)	(0.203)	(0.246)
Change × Majority			0.101
			(0.205)
Change × Minority × Lagged payoff	0.0260	-0.0169	-0.00675
	(0.0305)	(0.0233)	(0.0288)
Change × Minority × Lagged payoff			0.00386
			(0.0363)
round	-0.000960	-0.00827**	-0.00450
	(0.00188)	(0.00308)	(0.00292)
Constant	-0.982***	-0.884***	-1.335***
	(0.135)	(0.171)	(0.208)
Observations	8,910	8,689	8,689
Number of groups	30	30	30

Logit estimates with group clustered standard errors in parentheses; *** p<0.001, ** p<0.01, * p<0.05.

A byproduct of the payoff in the high conformity (HiConf) condition is a more nuanced social information compared to the low conformity (LoConf) condition. In the LoConf condition, players can only infer whether they are in the majority or minority in each round by comparing their individual choice to the group choice. However, in the HiConf condition players can also infer whether the group decision in each round is unanimous (3/3) or a majority decision (2/3) based on the exact payoff they receive. Specifically, the HiConf condition implicitly allows players to also know whether they are part of a majority decision (payoffs of -4 or 8) or a unanimous decision (payoffs of -5 or 7).

To assess whether this difference in social information available in the two group conditions might affect the differences found between the two conditions, we estimated the individual-level results separately for each condition in models 1 and 2. In model 3, we estimated the results for the HiConf condition while separating out majority and unanimous decisions, with the latter serve as the baseline category.

The main comparison is between the effect of social information (minority opinion) in the HiConf condition before and after the change in the game. The results show that in the stable stage of the game (rounds 1-60), being in a minority has a larger effect in the HiConf condition than in the LoConf condition. Model 3 shows that this effect (compared to a unanimous decision) is even larger, and being part of the majority also increased the propensity to change the subsequent choice compared to being part of a unanimous decision. Furthermore, the effect of being in the minority increased after the change in the game (rounds 61-100), as indicated by the estimated coefficients Change × Minority in Models 2 and 3. The effect of being in the majority (versus a unanimous decision) does not change, as shown by the small and insignificant coefficient term Change × Majority in Model 4. As our main individual-level findings suggest, being in the minority nullifies the effect of the payoff in the three models, but being in the majority in the HiConf condition does not alter the effect of the payoff, as reflected by the small and insignificant coefficient terms Lagged payoff × Majority in Model 3.

Note that the current models are separated by group condition whereas the models in Table 3 are separated by the stage of the game, and some of the interaction terms involved are different. This entails somewhat different interpretation of the coefficients in the two analyses (e.g., the coefficient "Social info" (minority) in Table 3 Model 6 estimates the effect of being in a minority opinion in the LoConf condition at the altered stage of the game with payoff=0. The same coefficient in Table A3 Model 2 estimates the effect of being in a minority opinion in HiConf condition, at the stable stage of the game with payoff=0.

These results show that the main findings of this study are robust to the more nuanced social information available for participants in the HiConf condition and are retained when accounting for these differences explicitly in the statistical analysis.
